# Kikuchi-Fujimoto Disease Following COVID-19 Infection in a 7-Year-Old Girl: A Case Report and Literature Review

**DOI:** 10.7759/cureus.26540

**Published:** 2022-07-03

**Authors:** Yusuke Saito, Yuta Suwa, Yakuto Kaneko, Mitsuhiro Tsujiwaki, Yasuhisa Odagawa

**Affiliations:** 1 Pediatrics, Otaru General Hospital, Otaru, JPN; 2 Otolaryngology, Otaru General Hospital, Otaru, JPN; 3 Clinical Pathology, Otaru General Hospital, Otaru, JPN

**Keywords:** kikuchi-fujimoto disease, histiocytic necrotizing lymphadenitis, cervical lymphadenopathy, sars-cov-2, covid-19

## Abstract

The coronavirus disease 2019 (COVID-19) symptoms in children are relatively mild and often do not require treatment. Nonetheless, complications caused by the immune response to COVID-19 in children are possible and diverse. We present the case of a 7-year-old girl with persistent fever and lymphadenopathy arising from SARS-CoV-2 infection, diagnosed with Kikuchi-Fujimoto Disease (KFD) on lymph node biopsy. KFD is a rare benign disease, clinically characterized by fever and tender cervical lymphadenopathy affecting posterior cervical lymph nodes. We also reviewed six previously reported cases of COVID-19-associated KFD that occurred in school-aged children and compared them with the present case. The clinical course of COVID-19-associated KFD was similar to that of previous reports of KFD with a favorable prognosis. This is the first report of a school-aged child developing KFD following SARS-CoV-2 infection. KFD should be considered when approaching patients with hyperinflammatory states who present with prolonged fever and cervical lymphadenopathy after COVID-19.

## Introduction

The coronavirus disease 2019 (COVID-19) pandemic caused by the severe acute respiratory syndrome coronavirus 2 (SARS-CoV-2) is still widespread. Its symptoms in children are relatively mild, without the need of treatment in many cases [1]. However, complications caused by the immune response following COVID-19 in children and neonates are diverse [2]. At present, COVID-19 is a disease that have the potential to lead to devastating sequelae as a result of an induced hyperinflammatory state. There are reported cases of multisystem inflammatory syndrome in children (MIS-C) and Kawasaki disease following COVID-19 infection in children [3,4]. Kikuchi-Fujimoto disease (KFD) or histiocytic necrotizing lymphadenitis, is a rare, generally self-limiting condition of unknown cause, usually characterized by cervical lymphadenopathy, fever, and leukopenia [5,6]. While the pathogenesis of KFD is unknown, the clinical presentation, course, and histologic changes suggest an immune response of T cells and histiocytes to an infectious agent. Infectious agents, including Epstein-Barr virus, cytomegalovirus, human herpesvirus 6, human T-lymphotropic virus type 1, rhinovirus, and parvovirus B19 were proposed to be predisposing factors for KFD [7]. In addition, a few cases of KFD after COVID-19 have been reported in adolescents and adults. We encountered a school-aged patient, who presented with fever and lymphadenopathy following SARS-CoV-2 infection that turned out to be KFD.

## Case presentation

A previously healthy 7-year old Japanese girl was admitted to our hospital with an 8-day history of fever and cervical lymphadenopathy following a SARS-CoV-2 infection. The patient had no history of respiratory symptoms. She was fully vaccinated and did not have any type of allergy. There was no recent history of travel, and contact with animals. She was initially diagnosed with lymphadenitis and treated with oral antibiotics (cefpodoxime proxetil) for three days and intravenous antibiotics (ampicillin-sulbactam) for three more days with no improvement. The complete blood count showed the following results: white cell count: 4.1 × 10^3^ cells/μL; absolute lymphocyte count : 2.1 × 10^3^ cells/μL; absolute neutrophil count: 1.7 × 10^3^ cells/μL; platelets: 212× 10^3^ cells/μL; and hemoglobin: 11.6 g/dL. The following biochemistries were also observed: D-dimer level: 1.4 mg/L; lactate dehydrogenase: 291 U/L; aspartate aminotransferase: 21 U/L; alanine aminotransferase: 11 U/L; triglyceride: 130 mg/dL; C-reactive protein: 3.1 mg/dL; serum procalcitonin: 0.12 ng/mL; ferritin: 160 ng/mL with negative antinuclear antibody. Finally, the cytomegalovirus and Epstein-Barr virus serology tests were compatible with past infections (Table 1).

**Table 1:** Laboratory investigations

Laboratory	Result	Reference range
Complete blood count		
White blood cells	4.1 x 10^3^/μL	3.3-8.6 (x 10^3^/μL)
Neutrophil count	1.7 x 10^3^/μL	-
Lymphocyte count	2.1 x 10^3^/μL	-
Hemoglobin	11.6 g/dL	11.6-14.8 g/dL
Platelets	212 x 103/μL	158-348 x 10^3^/μL
Biochemistries		
Lactate dehydrogenase	291 U/L	124-222 U/L
Aspartate aminotransferase	21 U/L	13-30 U/L
Alanine aminotransferase	11 U/L	7-23 U/L
Triglyceride	130 mg/dL	30-117 mg/dL
C-reactive protein	3.1 mg/dL	0.00-0.14 mg/dL
Procalcitonin	0.12 ng/mL	0.00-0.05 ng/mL
Ferritin	160.36 ng/mL	4.63-204 ng/mL
Erythrocyte sedimentation rate	70 mm/hour	3-15 mm/hour
Antinuclear antibodies	<40	<40
D-dimer	1.4 μg/mL	0.0-1.01 μg/mL
Human soluble interleukin 2 receptor	641 μg/mL	157-474 μg/mL
CD4	28.80%	24.3-49.7 %
CD8	33%	18.4-49 %
CD4/8	0.9	0.4-1.9
Cytomegalovirus IgM	0.13 Index	<0.85 Index
Cytomegalovirus IgG	161 AU/mL	<6 AU/mL
Early antigen-diffuse-IgG	<10	<10
Viral capsid antigen IgM	<10	<10
Viral capsid antigen IgG	40	<10
Epstein-Barr nuclear antigen	40	<10

**Figure 1:**
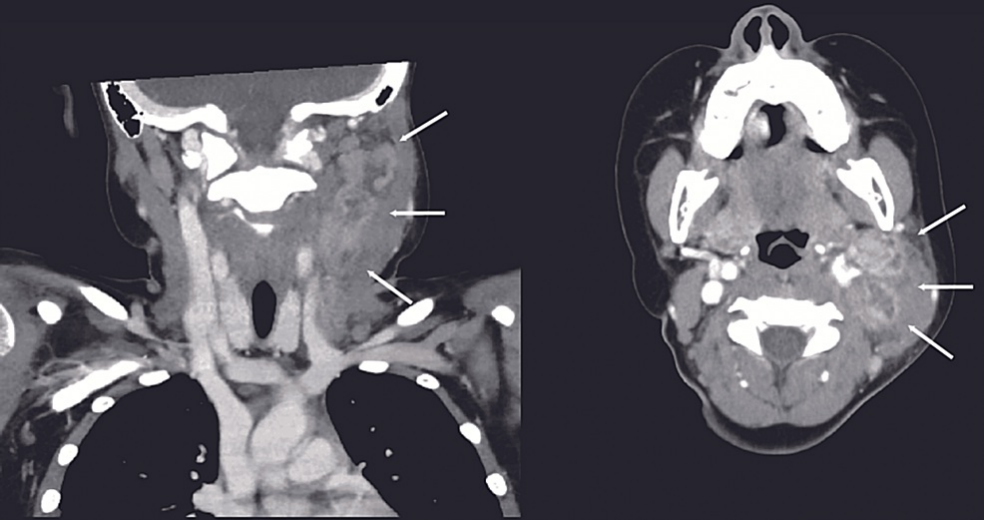
Coronal and axial cuts of a contrast-enhanced computed tomography of the neck Coronal and axial cuts of a contrast-enhanced computed tomography of the neck showing multiple enlarged and enhanced lymph nodes in the left cervical chain (arrows).

**Figure 2:**
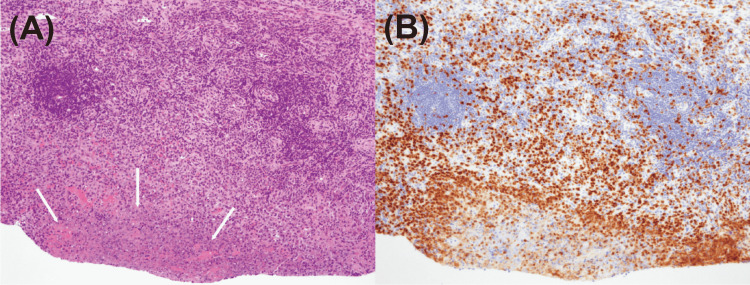
Histopathology of the lymph node biopsy specimens (A) Hematoxylin & eosin (H&E) stain (100× magnification) showing zones of necrosis. Necrotizing lymphadenitis with Kikuchi-Fujimoto disease-like features (B) Histiocytes are CD68-positive. (100× magnification)

A computed tomography revealed five left cervical lymph nodes measuring approximately 25 × 20 mm without enlargement of lymph nodes in the axillary, mediastinum, or thoracic chains. The lymph nodes showed a focal low attenuation, and a central nodal necrotic change was suspected (Figure 1). The biopsy revealed a lymph node architecture with diffuse polymorphic lymphohistiocytic infiltrate with foci of necrosis (Figure 2A). Immunohistochemically, we observed positive staining for CD68 histiocytes (Figure 2B) admixed with CD3-positive T cells and CD20-positive B cells. Lymphoid malignancy was not detected. The flow cytometry results were negative for monotypic B and T cells. The patient tested negative for the COVID-19 rapid antigen test on day 11, with defervescence on day 15. The skin lesions appeared on day 18 and resolved on day 25.

The lymphadenopathy also showed a tendency to shrink by day 28. Considering the course of the disease (fever, lymphadenopathy, and skin lesions), the results of the computed tomography, and histopathological examination of the lymph node, KFD was diagnosed and was attributed to a SARS-CoV-2 infection. Due to the spontaneous improvement of her general condition, normalization of laboratory tests, it was decided that no targeted treatment was necessary. The patient was discharged in good condition. Patients should be closely followed for recurrence.

## Discussion

Pediatric COVID-19 infections are mild and often asymptomatic. There is a low risk of severe illness or death in children with COVID-19 [1]. The cervical lymphadenopathy and fever for more than seven days are atypical in pediatric COVID-19 patients and requires scrutiny of the cause to select effective treatment.

More recently, six cases of KFD were described after COVID-19, the main features of which are listed in Table 2 [8-13]. This patient was the first school-aged child among the six reported cases of KFD after COVID-19 infection, with an age of onset ranging from 5 to 43 years. It has been reported that the duration of KFD onset after COVID-19 infection ranges from 1 to 3 months; however, only this patient had a fever that persisted after COVID-19. The clinical features, laboratory findings, and recurrence of KFD may differ according to age [14]. Fever, tender lymph nodes, and skin lesions are far more prevalent in children than in adults. Myalgia and weight loss were significantly higher in adults than in children [14]. COVID-19-related KFD showed the same clinical symptoms as those previously reported. This case had clinical symptoms typical of children compared to the previous five cases. A previous report showed that KFD patients presented with lymphadenopathy involving cervical, axillary, inguinal, and mesenteric nodes in 90.0%, 8.8%, 6.3%, and 2.5% of patients, respectively [14]. There was no difference in lymph node size between children and adults [14]. Cervical lymphadenopathy, often exceeding 2 cm in diameter, was noted in all cases. The clinical course of these cases was favorable, necessitating only symptomatic treatment to control fever.

**Table 2:** Main features of the cases of COVID-19-associated Kikuchi-Fujimoto disease LAD: left anterior descending; LN: lymph node; NSAIDs: non-steroidal anti-inflammatory drugs

Author	Gender	Age	The interval between the first symptom or lymphadenopathy and COVID-19	Clinical presentation	Site of LAD	LN maximum size (cm)	Treatment	Outcome
Stimson et al. [8]	M	17	Unknown	Lymphadenopathy, parotid gland enlargement, fever, weight loss, and fatigue	Cervical	1.3	No data	Complete resolution
Racette et al. [9]	M	32	3 months	Fever, chills, neck swelling, fatigue, myalgias	Cervical	No data	Prednisone	Complete resolution
Jaseb et al. [10]	F	16	Unknown	Lymphadenopathy, fever, night sweats, myalgia, weight loss, erythematous plaques	Cervical	2.5	Prednisone	Improvement
Masiak et al. [11]	M	43	5 weeks	Lymphadenopathy, fever, skin lesions, hepatosplenomegaly, cardiac involvement	Supraclavicular, cervical	2	Antipyretics	Complete resolution except heart function
Al Ghadeer et al. [12]	M	13	1 month	Lymphadenopathy, fever, night sweating, weight loss, anorexia, abdominal pain	Cervical	2.8	NSAIDs	Complete resolution
Öztürk et al. [13]	M	5	5 weeks	Lymphadenopathy, fever, sore throat	Cervical, axillary, inguinal	2.0-5.0	No treatment	Complete resolution
Presented case	F	8	Simultaneous	Lymphadenopathy, fever, skin lesions	Cervical	2.5	No treatment	Complete resolution

The most common hypotheses discussed in the literature are infectious and autoimmune conditions, which may manifest similarly. Several infectious agents were supposed to incite KFD including Epstein-Barr virus, human immunodeficiency virus, human herpesvirus 6, human T-lymphotropic virus type 1, and parvovirus B19. However, there is no evidence that infection may directly cause KFD, and several studies have failed to detect these infectious agents in the involved lymph nodes [7]. To investigate the association between KFD and COVID-19, an attempt was made to detect SARS-CoV-2 in lymphoid tissue in one case; however, the result was negative [9]. In addition, five cases of KFD after COVID-19 vaccination have been reported [15-18]. These reports suggest that the immunological mechanism induced by COVID-19 cause KFD.

With no other cause of KFD other than COVID-19 in the patient, the diagnosis was confirmed by CT findings and histopathological examination. The histopathological findings were characterized by necrosis and the presence of T lymphocytes, including CD68+ histiocytes, CD4+ and CD8+ T cells, in agreement with previous reports [19,20]. Although inflammation is presumed to be involved in the pathogenesis of KFD after COVID-19, further analysis is required. Therefore, KFD should be considered in children with prolonged fever and lymphadenopathy after COVID-19. Patients should be closely followed for recurrence.

## Conclusions

We present the case of a 7-year-old girl with persistent fever and lymphadenopathy arising from SARS-CoV-2 infection, diagnosed with KFD on lymph node biopsy. The interval between the COVID-19 infection and KFD onset was at least one month in previous case reports, whereas the simultaneous existence of lymphadenopathy in this case clearly suggests that COVID-19 can be a direct cause of KFD. Pediatric COVID-19 infections are mild and often asymptomatic. However, KFD should be considered in children with prolonged fever and lymphadenopathy after COVID-19.
